# Multiple comparison correction methods for whole-body magnetic resonance imaging

**DOI:** 10.1117/1.JMI.7.1.014005

**Published:** 2020-02-28

**Authors:** Eva Breznik, Filip Malmberg, Joel Kullberg, Håkan Ahlström, Robin Strand

**Affiliations:** a Uppsala University, Centre for Image Analysis, Division of Visual Information and Interaction, Department of Information Technology, Uppsala, Sweden; b Uppsala University, Section of Radiology, Department of Surgical Sciences, Uppsala, Sweden; c Antaros Medical, Mölndal, Sweden

**Keywords:** whole-body magnetic resonance imaging, statistical analysis, multiple comparisons, correction methods, Imiomics

## Abstract

**Purpose**: Voxel-level hypothesis testing on images suffers from test multiplicity. Numerous correction methods exist, mainly applied and evaluated on neuroimaging and synthetic datasets. However, newly developed approaches like Imiomics, using different data and less common analysis types, also require multiplicity correction for more reliable inference. To handle the multiple comparisons in Imiomics, we aim to evaluate correction methods on whole-body MRI and correlation analyses, and to develop techniques specifically suited for the given analyses.

**Approach**: We evaluate the most common familywise error rate (FWER) limiting procedures on whole-body correlation analyses via standard (synthetic no-activation) nominal error rate estimation as well as smaller prior-knowledge based stringency analysis. Their performance is compared to our anatomy-based method extensions.

**Results**: Results show that nonparametric methods behave better for the given analyses. The proposed prior-knowledge based evaluation shows that the devised extensions including anatomical priors can achieve the same power while keeping the FWER closer to the desired rate.

**Conclusions**: Permutation-based approaches perform adequately and can be used within Imiomics. They can be improved by including information on image structure. We expect such method extensions to become even more relevant with new applications and larger datasets.

## 1 Introduction

With the advance of imaging technologies, whole-body magnetic resonance imaging (MRI) has become fast enough to make its use in research and even medical practice feasible. As the image acquisition time is shortened, this form of examination has begun to play an indispensable role in diagnostics for exploratory searches as well as tumor detection, staging, and therapy evaluation.^1^

Successful utilization of whole-body MRI in oncology applications prompts the question of possible uses in other areas with fundamentally different pathologies, for example, for systemic diseases, such as metabolic syndrome and diabetes,^1^ that are known to involve the whole body. Despite the valuable insights, however, there are potential pitfalls of using whole-body scans in practice.

The decision for medical intervention is usually based on detecting parameters that deviate significantly from what can be expected for a healthy subject. With regard to imagery, that means a certain amount of processing (using healthy patient data) aimed at detecting where significant deviations occur. This is especially true for Imiomics analyses,^2^ where additional subject-specific measurements are merged with image data, resulting in numerical maps, with values that are possibly more informative and comparable among the population than the raw intensities, such as, for example, correlations.

Depending on the targeted inquiries, the imaging data need to be analyzed statistically, which is typically done following a massive univariate approach, meaning (in terms of images) that at every individual voxel, a distinct analysis is carried out. In practice, this comprises conducting one statistical hypothesis test per each individual image unit (voxel) separately but nonetheless simultaneously. It produces a map of p values, representing the likelihood of observing given values, under the assumption of some particular distribution for the null case of no activity in the image.

Given a significance level (probability of a false discovery we are prepared to tolerate), using it to threshold the p values leaves us with a binary map indicating significant voxels, knowing that at each apparently significant (also positive, active) one, the probability of an erroneous detection is less than or equal to the chosen significance level. From here on, we use the term “activation” in voxel sense to denote deviations (either true underlying or just detected) from the statistic of interest in any given analysis. A more formal summary of the theory can be found, e.g., in Refs. 3–5.

However, when working with images, the whole image tends to be considered as one single entity and we want to be able to draw sensible conclusions on accuracy of the entire family of tests it comprises. The error rate of interest is therefore usually the number of erroneous positives (i.e., type I errors) over the “whole image,” which is the so-called familywise error rate (FWER). Unfortunately, knowing that each individual active voxel is wrongfully detected with at most probability p does not impose the same upper bound on FWER. Even worse, the more tests we conduct (i.e., the more voxels we have), the closer the probability of making at least some erroneous detection in the image is to 1.

This multiple comparison problem is a well-known problem in statistics, present wherever multiple simultaneous hypothesis tests are carried out, and as such common to all voxelwise statistical analyses on images. A very illustrative example of how not dealing with this problem can have disastrous consequences on the interpretation of the data is the study of a dead salmon in Ref. 6, where Bennett et al. note how ignoring the problem led to the presence of activity in the dead, obviously inactive brain. Moreover, some meta-analyses have been performed on published research in human neuroimaging, which as well point to inflated detection rates in the literature.^7^

Throughout the years, many approaches (called “correction methods”) have been developed in hopes of resolving this problem,^3^^,^^4^^,^^8^ but they depend heavily on the particular data in question, and failure to comply with their assumptions can again lead to inaccurate results.^9^^–^^11^ For that reason, multiplicity represents a pressing, omnipresent issue that should not go overlooked, and the correction to be used should be chosen carefully, depending on the properties of the data used in the specific analysis. While there is an open call for focusing on better statistical models and data acquisition instead of the raw p values, corrections, and significance,^12^ the use of correction methods is still the standard way of dealing with multiplicity issues and is therefore where the focus of this paper lies.

Most of the research on the development of new methods, as well as on applicability and effectiveness of already available ones, have been targeted at neuroimaging data. However, with the emergence of new concepts and applications of image analysis (such as Imiomics from Ref. 2), there is a need to evaluate the methods and establish some general consensus/guidelines for the case of whole-body MR data, too. Not only are there differences in anatomical and image properties, which already imply a possibly different behavior of the methods in the two cases but also in the types of analyses of interest. Correlation analyses, for example, are mostly avoided in neuroimaging due to small datasets. With whole-body MRI on the other hand, correlation can be particularly interesting from research perspective and studies often adequately powered for such analyses.

In this report, a number of methods, controlling the FWERs (at various inference levels; see Ref. 13) that are most frequently used in practice or tend to perform best with functional MRI (fMRI)^9^^,^^14^^,^^15^ are examined in the context of whole-body MR images: Bonferroni type correction,^16^^,^^17^ random field theory (RFT),^10^^,^^18^^–^^20^ basic permutation-based methods^10^^,^^21^ and threshold-free cluster enhancement (TFCE),^22^ as well as the cluster-based analysis (CBA)^23^ procedure with several choices of correction. In addition, we explore the possibility of using anatomical information as a basis for correction.

### 1.1 Contributions

The main contribution of this report lies in the evaluation of a variety of methods on a real-life dataset in search for a principled correction procedure, as well as in the introduction of a different evaluation pipeline.

Furthermore, possible ways of method evaluation are discussed, as the standard approach calls for a particular (no-activation) type of data that may be impossible to acquire. To the best of our knowledge, this is the first account of correction method validation on large-scale imaging data, outside neuroimaging applications, and consequently the first attempt at establishing a general validation case for it, analogous to the no-activity scan of the fMRI. The need for such method validation comes from the differences in anatomical and imaging properties of datasets from within neuroimaging and, e.g., from Imiomics, where whole-body MR scans are used. In addition, we focus on correlation analysis, which is rarely done in neuroimaging.

We also lay grounds for introducing more anatomy-aware methods of correction, specifically tailored for our problem at hand. There have been attempts of adding other biological priors (see Ref. 24), but the present article represents the first account of direct inclusion of large-scale, spatial anatomical information in the multiplicity correction.

## 2 Methods and Materials

We first cover previous work on the topic by briefly describing the mechanisms of the methods we used in this study to facilitate the subsequent reasoning behind their potential pitfalls. Section 2.1.2 then introduces a simple idea for a novel approach based on prior information on our whole-body data and shows some theoretical implications of applying this method and method extensions. To test the appropriateness of different methods for whole-body scans and Imiomics analyses, we use medical data from the prospective investigation of obesity, energy and metabolism (POEM) cohort. A discussion on evaluation strategies is provided in Sec. 2.2, followed by a detailed description of the dataset in Sec. 2.3.

### 2.1 Correction Approaches

Different methods can control different overall error rates,^14^^,^^25^ for example, the FWER, false-discovery rate (FDR), false-discovery proportion (FDP), per comparison error rate, etc. And they can also work at various inference levels,^13^ meaning in essence that they treat various amounts of the input imaging data as individual entities in the inference step (e.g., individual voxels, clusters, sets).

The majority of the correction methods in wider use within fMRI and neuroimaging, in general, are designed to limit the FWER error, which is the probability of at least one error being made in the whole family of tests. They have different assumptions on data properties and based on the extent of compliance with them, they result in various stringency levels. In recent years, a lot of effort has been put into developing the FDR limiting procedures as well,^26^^–^^28^ as they tend to be more stable and less stringent. However, when using the methods in medical practice, where presence of a signal at individual voxels (or structures containing only small or varying amounts of them) is of importance, focusing on an expected rate of errors can hinder confidence in the medical interpretation of the results.

Available evaluations of multiple comparison correction methods for integrated imaging data deal, to our knowledge, exclusively with neuroimaging data; the most comprehensive evaluation of methods (as well as widely used software tools implementing them) on imaging data to date, namely using fMRI, was done in Ref. 9. Another extensive evaluation of cluster-based correction, though only of permutation-based methods, is also available in Ref. 15. For a simulation-based evaluation of the basic correction methods (for both FWER and FDR) on data with varying degrees of positive dependence among tests, see Ref. 29.

#### 2.1.1 Controlling FWER—previous work

The following methods are explained briefly, and the interested reader is encouraged to explore the original references: Bonferroni-style step-down procedures,^16^^,^^17^ RFT,^18^^–^^20^^,^^30^ permutation-based methods,^21^^,^^22^^,^^31^ CBA,^23^ methods for generalized FWER control,^32^^–^^34^ and parametric-bootstrap joint (PBJ) testing.^35^

The simplest and oldest method for correction is the Bonferroni method,^16^ adjusting the significance level based only on the number of tests performed. It sets the same significance level for thresholding at all voxels and corrects it via division by the number of tests. The assumptions behind it are that all the tests are independent, which is not really the case in images, where some spatial correlation is almost universally present. The violation of independence still results in theoretically valid but extremely stringent thresholds that tend to also wipe out the true active signal. Regardless, it is still used a lot in different fields due to its simplicity.

A simple yet powerful extension of the basic Bonferroni method is the Holm step-down procedure from Ref. 17. The difference between them is that Holm’s method rejects the tests sequentially, each one at different thresholds, depending on their order with respect to signal size. This way it requires more evidence toward rejections for those values that are less extreme. And only the last value’s threshold for significance is lowered by the factor of all tests, which is in contrast done for every value in the simple Bonferroni version. There are further extensions to the Bonferroni method available (for example, Hochberg procedure from Ref. 36) but are omitted here as the stringency (of Bonferroni-type corrections in general) is very high compared to other types of correction procedures.

Random field theory,^11^^,^^18^^,^^19^^,^^30^ built upon the notion of Euler characteristic (EC) in a thresholded image, has been most popular within the neuroimaging community in recent years. Using the expected value of EC, it derives a closed-form approximation for the tail of the null distribution for the voxel as well as for cluster-based correction. It is thus computationally undemanding but the mathematical theory behind it, on the other hand, is quite sophisticated. It was developed as a solution to spatial correlation problem of Bonferroni-like procedures, but it introduces other restrictions, such as sufficient smoothness of the image, twice-differentiable autocorrelation function, and the same parametric distribution independent of the spatial location; all are often untenable for our images in practice.

In contrast to simply assuming a certain null distribution for the statistic, it can also be estimated from our data. This is the fundamental idea behind the permutation (resampling based) methods:^21^^,^^22^^,^^31^^,^^37^^,^^38^ randomly permute the data numerous times, calculate the extreme values of the chosen statistic under the given permutation, and build an empirical distribution from those values. The FWER corrected p values at a given significance level α are then obtained as quantiles of this empirically calculated distribution. Such methods are nonparametric and do not require the data to satisfy the complicated assumptions of RFT for validity. The only requirement that needs to be met is the exchangeability of subject/parameters that are being permuted (and usually, aiming for distributional symmetry is enough). In neuroimaging, these permutation-based joint testing procedures appear to be the only ones that can reliably control the FWER^9^^,^^38^^,^^39^ and have therefore been the basis for most of the latest development. To relax even the exchangeability requirement, for example, and to speed up the execution (which is their major drawback), a version of the method called PBJ testing procedure has been proposed in Ref. 35. Another possibility for speeding up the permutation tests is doing approximate inference, as presented in Ref. 40.

Apart from the standard voxel signal, cluster size, and cluster mass statistics, such permutation approaches can also be used for acquiring the distribution of a more complicated statistic, encoding signal strength as well as spatial extent information. One example of such a method is called TFCE,^22^ and the statistic it uses, the TFCE-score, is a voxel-based measure. This way, there is no loss of localization power while the spatial extent is still included in the calculations through the integration of spatial support for the given voxel over a set of thresholds. More recently, a probabilistic version of TFCE has been proposed,^41^ which reuses the TFCE idea but avoids the time-consuming permutations in order to speed up the execution.

Traditionally, when aiming for cluster-based inference,^18^^,^^21^^,^^30^ the cluster formation is based on the raw voxel signals and a cluster-defining signal threshold that can be chosen arbitrarily. Since the appearance of the so-acquired clusters is very sensitive to the choice of this threshold, it is important to keep in mind that even slight changes in its value can have unprecedented effects on the final p values. Another possibility would be to instead prepartition the image into clusters, assign them a signal value via some summarizing statistic (e.g., mean, maximum), and run any chosen voxel-based correction method, treating the clusters as new units for correction. The CBA as proposed in Ref. 23 does exactly that; it predefines clusters based on spatial covariance and runs an adaptive procedure to control FDR on them (but one could just as well run a simple Bonferroni-type correction on the clusters as units, to control the FWER). The idea behind the clustering is to group each voxel together with the neighboring voxel with which it is most highly correlated, where correlation coefficients for neighbors sharing only an edge or a vertex are corrected for the larger grid distance with respect to the direct neighbors sharing a face. The cluster signals are simply the averages of the contained voxel signals. This results in very small clusters that should not negatively affect the visual interpretation of the image but are nevertheless still large enough to at least halve the multiplicity burden.

The notion of familywise error can be generalized to allow for additional mistakes by the FDP,^32^ denoting the ratio of false discoveries among all of the discovered voxels. The expected value of the FDP, E(FDP), is actually the so-called FDR,^42^ which is more widely known and used in the literature. But since FDR represents an expected value of the error on average, its control does not imply control in every individual experiment; in other words, the actual FDP is still not prohibited from varying, meaning that the FDR limiting procedures are not applicable at least for use in medicine when the exact knowledge on the upper bound of the error rate is desired for each specific experiment, which is why they are out of the scope of this paper.

#### 2.1.2 Controlling FWER—proposed anatomy inspired corrections and extensions

Since our aim is to test the methods on whole-body scans, which carry specific expected anatomy, it seems natural to try and include this information in the corrections. However, as most of the multiple comparison correction development has been targeted at fMRI, where structures are less well defined, not much work has been done in this direction.

The way prior spatial anatomical information has usually been added in previous work is by defining smaller regions of interest (ROIs), and thus excluding large parts of images (tissues and organs), where the activation is not expected to be of interest (for example, Ref. 43). That effectively lowers the multiplicity burden as a preprocessing step, and thus improves the results of whichever correction method is applied in the end.

While focusing on a specific ROI is an easy, perhaps most straightforward way of alleviating the multiple comparison problem, it is not always applicable. Whole-body scans are very often used as exploratory scans, in cases where no good assumption regarding the locality is available, and therefore, the anatomy has to be included in some other way.

To our knowledge, the only method that aimed for the inclusion of anatomical information without limiting the correction to a specific region of interest is the one described in Ref. 24, which was developed explicitly for neuroimaging using a brain-specific prior of hemisphere symmetry.

We propose some possible directions toward a more structured, anatomy-compliant correction, which offers additional power with the use of the prior data though it also suffers from a similar drawback as the ROI focused improvement: uncertainties that stem from extracting the anatomical priors. As those are a prerequisite for the methods we develop in this paper, we hereon assume a reasonably accurate segmentation of all tissues in question is available.

##### Limiting FWER on predefined anatomy-based clusters

When working on research questions involving organ activity, where the activity is, if present, most likely to be found over the whole organ area, it follows intuitively that treating the organs as units is a good way of alleviating the multiplicity problem. For this, however, all the delineations of the organs, tissues, or grouping regions must be known, and activity can in the final step be inferred only for those whole regions.

Given a segmentation method with a known accuracy (either an automatic tool or a number of manual segmentations) per organ, and a proper summarizing statistic for the cluster signals, we can effectively limit the FWER on the cluster level by using, e.g., the Holm procedure. The question here is how to devise a summarizing statistic for cluster signals that would allow us to boost the signal-to-noise ratio while somehow including the uncertainty of cluster membership at each voxel.

While averaging over the organs could potentially help with SNR, it is also sensitive to including a border of voxels that do not belong to the organ in question. Since the segmentations we can acquire are never completely accurate, we need to compensate for those errors. We propose using a weighted average for cluster signals, where weights can be assumed to decrease toward the edges of the segmentation. This can be done by using a soft segmentation, joining labelings from a number of annotators (i.e., if n out of N annotators label a voxel as liver, and the other N−n as kidney, than the weights with which the signal of that particular voxel contributes to the liver and kidney cluster signals, respectively, are $\frac{n}{N}$ and $\frac{N-n}{N}$).

To formalize, let $S_{k}$ denote the signal of the cluster representing some structure $C_{k}$, k∈{1…K}. $x_{i}$ the image signal at voxel i and $U_{k}$ union of all voxels that have a nonzero membership for cluster $C_{k}$ (or where the accuracy of the binary cluster-background segmentation is nonzero) in any of the annotations. Then, the signal of a cluster is calculated via the following weighting average:

$$S_{k}=\frac{1}{\left|U_{k}\right|}\underset{i\in U_{k}}{\sum }x_{i}\cdot a_{k}(i),$$

where $a_{k}:U_{k}\to R$ is the accuracy function for the given structure cluster. In the case of N available annotations, we then have $a_{k}(i)=\frac{n_{i}}{N}$ with $n_{i}$ the number of those annotations that label voxel i as belonging to a cluster of structure $C_{k}$. Since gathering multiple annotations is not always feasible, a way to approximate such an accuracy map $a_{k}(\cdot )$ (unless it can be directly produced by the employed segmentation method, e.g., when using neural networks) is to assume thin border around the boundary of each segmented organ having lower reliability.

Finally, to ensure the FWER control, permutation distribution can be acquired for the defined cluster/organ statistic and the resulting p values can be adjusted via, e.g., the Holm procedure (now the multiplicity is low, as only K simultaneous tests were performed and in addition, the tests tend to be more independent—so, the stringency of this type of procedures is not an issue anymore). The interpretation of the final p values is then similar to the voxelwise p values: p value of an organ X with weighted signal S denotes the probability that under the assumption of no activity in the body, a signal equal to or exceeding S is present in X.

In theory, we could do the correction on the newly defined statistic directly through the maximal distribution by permutation. However, the statistic values would then need to be somehow normalized to be comparable among organs, which can sometimes be difficult to achieve (different organs can have different amounts of voxels with varying quality or label confidence).

##### Using anatomical information within CBA, TFCE, and permutation methods

When applying the established approaches, anatomical information can sometimes be included via the specific choice of parameters. For example, using k-FWER, expected sizes (in voxels) of active clusters (e.g., corresponding to sizes of organs) could be included to some extent by setting their minimum (or a certain fraction of it) as an upper bound for k. This way we allow as many errors as possible without hindering the final (per-organ) inference on activity.

This particular example utilizes very little of the organ cluster knowledge (in essence only the size of the smallest one), however, sizes of all individual objects can be included within CBA and permutation-based (also TFCE) methods by imposing anatomical restrictions to the support growth and clustering, respectively. Naturally, such extension has some implications for TFCE score due to a large variability in organ size. For example, with the generic choice of TFCE method parameters, because of the liver being larger than the kidney, its overall signal can be much lower than the one of the kidney, for both to be detected as active. The workaround for this is to adapt parameters, giving less power to the size of clusters or scale size by the maximal possible one (so that a three-voxel signal inside a pancreas of nine voxels is considered as extensive as a 15-voxel signal in a liver consisting of 45 voxels).

The modified TFCE score for the case of including anatomical extent knowledge is then equal to

$$\mathrm{TFCE}(x)=\overset{T}{\underset{t=t_{0}}{\sum }}{\left(e(t)\cdot \frac{e_{\max}}{e_{X}}\right)}^{E}t^{H}dt,$$

where x∈X, $e_{X}$ means the extent of the organ X, and e(t) is now the extent at threshold t inside the organ or structure X (instead of the whole picture). And $e_{\max}=\max_{X}\;e_{X}$ is the size of the maximal object. The parameters E and H that are carried over from the original TFCE method formulation represent the importance of signal extent and strength, respectively. These can be tuned depending on what is considered more indicative of true activation in the specific setting.

By dividing the signal extent over organ by the maximal organ extent, we ensure that a spatially smaller activity in a small structure is treated similarly as a spatially more extensive activity inside a larger object. This, however, means that there will be trouble when trying to detect small partial activities in larger structures, but that is the cost we may be prepared to pay to alleviate the problem of unequal object sizes, particularly when activities are expected to span the whole organs or larger parts thereof. It also does not explicitly sanction the extents that span over the organ borders but might just be smeared activity (instead of an actual several organ spanning one). This goes along with the inherent assumption that the activity outside one organ belongs to another structure (even if contiguous with the active voxels in the first structure).

Another modification that can be made to account for the anatomy is the clustering in the CBA method. As explained above, the method clusters the base image (not used in the analysis) depending on the correlation between voxels (taking into consideration also their distances). In such a clustering, we can expect to obtain smaller clusters. Due to image artifacts, however, it can easily happen that the clusters span over multiple organs. Once again, we can restrict their growth during clustering. The anatomy-preserving version of the CBA clusters can then have a much different appearance, particularly close to the borders of the individual organs. Depending on how we conduct the clustering process (it is sensitive to choice of the starting point), we might also produce more smaller clusters, with possibly more spiked signals (since their signal is averaged over a smaller surface).

Within the permutation-based correction, the anatomical prior can be included in a similar way as above. If the activity extent is of interest, we can carry out the original cluster-extent permutation-based correction, but again limit the growth of clusters to within individual organs. As with TFCE, the obtained signals need to be scaled to the organ sizes since the permutation-based correction works by noting the maximal statistics (here extent) over the body and thus requires the individual organ-limited cluster extents to be comparable in size.

This is very similar to the method described in the previous paragraph. The difference, however, is that there the signal is defined over the organ and thus the p values and significance can be determined on a structural level. Here, one organ can contain multiple sources of activity. While one can decide whether to join them or use them separately during the permutation distribution calculation, the resulting p values still correspond only to the voxels of activity, included in the computation, and not the entire organ. This way we retain more spatial locality information about the activity.

The interpretation of the acquired p values is now slightly different than that of the original methods. Then, we were interested in the probability of an activity-cluster of at least some given extent appearing under the null hypothesis, whereas now the probability in question is that of a cluster spanning at least some given fraction of any organ (again, given the null hypothesis is true).

### 2.2 Method Evaluation Strategies

In fMRI, the methods can usually be evaluated directly in terms of type I error on the resting state image sequences. In whole-body images, however, we do not possess proper rest-state, no-activity images. Due to this, we shall evaluate the methods in two ways: on an approximation of null activity case for the correlation analyses and by empirically evaluating the type II error on images with known activity.

The latter approach to evaluation is perhaps not intuitive, as it deals with another error rate. It is known, however, that the type I and type II errors are related^44^ and that bounding one can increase the other. The exact interplay is, on the other hand, not explicitly known as it depends on the distribution of possible alternative hypotheses. For that reason, even when comparing the methods that theoretically should impose the same upper bound on the type I error, looking at their type II error rates can pay off. Under the assumption that the methods work as they should (i.e., truly keep the false positives at a known level), the appropriateness of their application can be measured by their effect on truly active data; the more sensitive the method appears to be on the data at hand, the better.

On top of that, sensitivity can sometimes be more important than specificity in medical applications;^44^ for exploratory searches, for example, which are intended to provide a pool of possible diagnoses, identifying potentially pathological activity is more important than being particularly exact about its extent and precise location, especially when final diagnosis is not based entirely on images but rather supplemented with subsequent, more targeted confirmation strategies. In addition, with such large number of voxels and additional medical knowledge, it is much easier to find a case where we know from theory that some activation should be present than it is to find an analogous case for which we can with all certainty claim there should be none, so a testing case of such type could be easier to come by.

For the first approach to evaluation, we use an artificially constructed null-case image for the correlation analysis. It is constructed by using a random vector for calculating the correlations with voxel intensities. Considering the high number of voxels, many spurious responses can be expected. And the extent to which the methods are able to remove those activations is considered as the evaluation metric in two ways: similar to Ref. 9, we can observe the nominal error rates after a number of analyses, but we can also look at a few example analyses to see not only whether the errors are present or not, but rather how prevalent the errors are in the final results. This can, together with the known-activity results, be somewhat indicative of the stringency level and represent important information when we are more concerned with the possibility of an erroneous interpretation than individual voxel errors. Nonetheless, this way of constructing a no activity map is not entirely equivalent to the fMRI no activity scan situation, where the responses under the null hypothesis are actually spurious and occur due to different noise sources. In our case, however, the construction of the random vector does not necessarily imply that the null hypothesis holds at every voxel. Thus, it may happen that the amount of activations after correction is higher than theoretically expected.

We then look at an example amounts of spurious activity that survive the correction as well as at nominal error values over 200 experiments.

For the second evaluation strategy, we need a reference true activity map. As in Ref. 45, this could be done by first running the analysis on a much larger sample (in order to ensure reasonable power) and then using the acquired results as the reference. While such a procedure can lead to a quite reliable reference given a reasonable number of samples, it requires a large amount of samples to create the reference, and the reference still needs to be corrected for multiplicity before use. For this reason, we here instead resort to using a combination of analysis and data for which we have some prior knowledge on the true activity. We chose a correlation analysis with bioimpedance analysis (BIA, a measure of body fat mass) as the nonimaging parameter. It represents the total amount of fat within the body, which, of course, correlates highly with the fat tissue in the fat/water separated MR images. This enables us to approximate the amount of truly correlated voxels that are retained after correction. The voxels with a sufficient amount of fat to be used as truly active voxels in the evaluation are acquired by thresholding the fat image of the reference subject (the same reference as used for the registration).

### 2.3 Dataset

The correction methods in this paper are evaluated using the POEM cohort (Ref. 46, PI: L. Lind, see Ref. 2 for more detail), containing whole-body MR scans of 342 subjects. Ethical approval for the study was obtained from the Regional Ethical Review Board in Uppsala, Sweden (Approval numbers: Uppsala Dnr 2009/057 and Dnr 2012/143), and written consent was obtained from all subjects. Scans were acquired on a 1.5T MR system with a whole-body water-fat imaging protocol using a spoiled 3D multigradient echo sequence and scan parameters TR/TE1/ΔTE=5.9/1.36/1.87  ms, three unipolar echoes, and 3 deg flip angle. The images are of size 256×252×256, reconstructed voxel size 2.07×8.0×2.07  mm, and were corrected for intensity inhomogeneities by slice-wise normalization of intensity values in the foot–head direction (to avoid discontinuities between adjacent axial slices) and simultaneous correction, as in Ref. 47.

They are all registered to a common coordinate system by a nonparametric three-step registration method,^2^ accounting for the elasticity of the tissue. The registration procedure provides Jacobian determinants (JDs) of the displacement fields that can be considered a measure of volumetric change between subjects. It is, however, accurate down to 2 pixel resolution, which motivates the decision not to use the segmentation boundaries in the evaluations (see Sec. 3 for more details). It could also be used as a reasoning for smoothing that is or should be done for certain methods prior to correction.^11^

In addition to the scans, medically relevant parameters, such as BIA, triglycerides, genetic traits, etc., are given for every patient, such that correlation maps can be calculated to measure the level of interconnection between various pieces of information. The correlation analysis in our experiments includes BIA measures and the JD values. The reasoning behind this choice is the knowledge we have on expected significant correlations: as BIA represents the total amount of fat in the body, and the JD shows relative volume, we expect the voxels in the fat tissue to exhibit high levels of activation while testing for significance. This enables us to directly (even visually) assess the stringency of the methods.

The subjects in the dataset are of both genders (approximately equally represented in the sample), 50 years of age. Possible confounding effects from age variation are therefore excluded, and given a relatively high number of data available, we avoid the gender effects by carrying out two separate analyses. As we are interested in negative as well as positive correlations, we focus on two-sided tests in our experiments.

In order to follow the described evaluation strategies and to implement the anatomy-based corrections, a reference segmentation is also provided for the male and female reference subjects. It includes around 48 separately labeled structures, including splitting of the fat tissue based on the more relaxed definition of body parts/areas. More specifically, the categories are the following: bladder, bowels, liver, pancreas, spleen, stomach, lungs, heart and large vessels, eyes, brain, and spinal cord. The muscles, bones, and fat tissue are all in separate categories, split according to body areas: calves, thighs, gluteus, abdominal area (fat here separated into SAT and VAT, and muscles on abdominal and back), upper back and shoulder area, and head. Semantic segmentation, particularly on a lower resolution MRI, can be very hard, so we add a tissue category other in which we join all those voxels that were too uncertain to be labeled as any of the original structures. Since voxels of this category appear at various spatial locations in the final segmentation, we split this category based on the general body area as well.

**Fig. 1:**
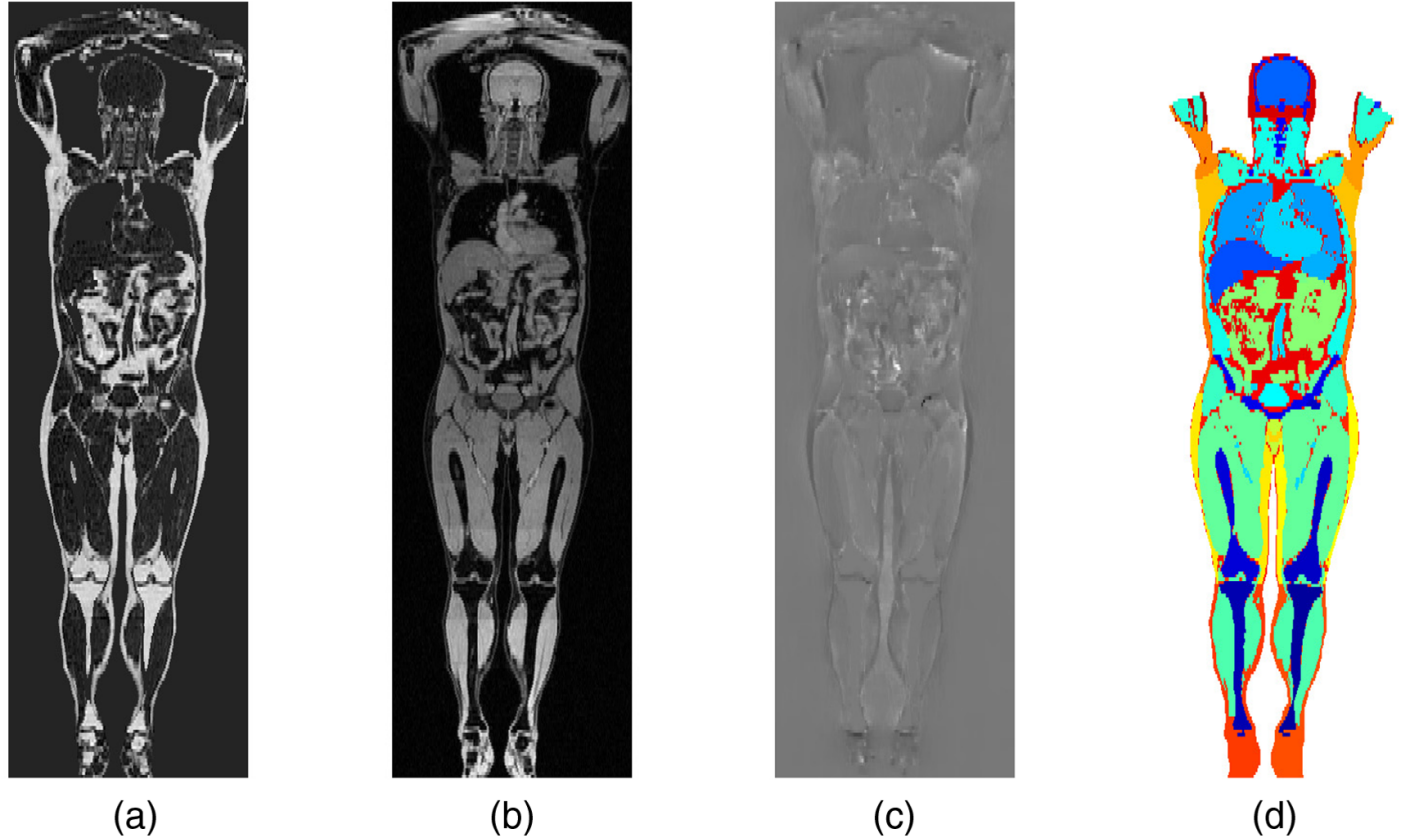
Example image slices for male subjects. (a), (b) The original MR fat-water separated images, showing (a) fat content and (b) water content. (c) The JD of the same slice and (d) the coarse semantic segmentation of the tissues and body parts.

Examples of all the images in use are shown in Fig. 1.

## 3 Results

**Fig. 2:**
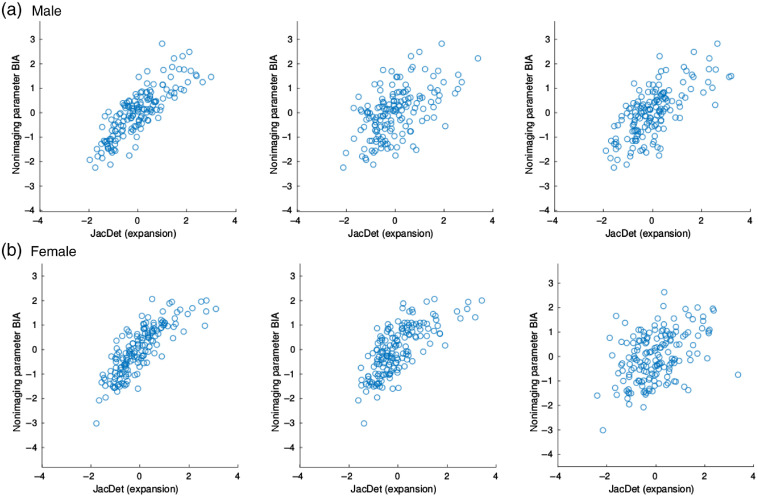
Example scatter plots for correlation between BIA measurements and the JD at a few voxel locations (chosen randomly from the areas where strong correlation is expected), shown for (a) male and (b) female subjects separately. We see that the exhibited correlation is approximately linear and that the data do not seem to suffer extensively from heteroscedasticity.

As mentioned above, for evaluating the stringency of methods, we use a combination of JD images and nonimaging measurement BIA to produce correlation maps with expected activity (which here means significantly large correlation values) in the fat tissue. Pearson’s linear correlation coefficient was used to measure correlation. We opted for the given choice after visually examining a few data points (see examples in Fig. 2) and noticing that the occasionally exhibited correlation was mostly linear in nature and that the assumptions for using the Pearson coefficient are satisfied—absence of outliers, approximately normal data distributions for both variables. In addition, the examination of variables at individual voxels shows that there is no prevalent residual heteroscedasticity, so we avoid the need to account for it in the evaluation.

The same correlation coefficient was used in both evaluations, on the original as well as with the null data. For the null case, we constructed a random vector of measurements (to be used in the correlation calculations instead of BIA), distributed according to a standard normal distribution. We chose the widely used significance level of α=0.05 and where needed, a cluster defining (primary) threshold corresponding to α=0.001. The parameters used within the TFCE method were the ones suggested in Ref. 22: H=2, E=0.5, and dt=0.1. For the proposed simple per-organ method, we assumed (equally) strong confidence for all the inside voxels (weight of 1) and a low one in the outermost border voxels (weight of 0.1). The correlation-based clustering that is part of the CBA method was carried out on a reference fat-content image, which was not included in the analyses.

### 3.1 Evaluation on Data with Activity

To properly evaluate the methods and their stringency, we approximate the amount of true positives as the amount of voxels with sufficiently high-fat content in the reference subject image; i.e., those that were segmented as subcutaneous fat. This value is, of course, not entirely accurate but serves the purpose of comparison and gives the feeling on method stringency. In order to account for the uncertainty in the segmentation, we also exclude the outer boundary in the counts affecting the final evaluations.

**Fig. 3:**
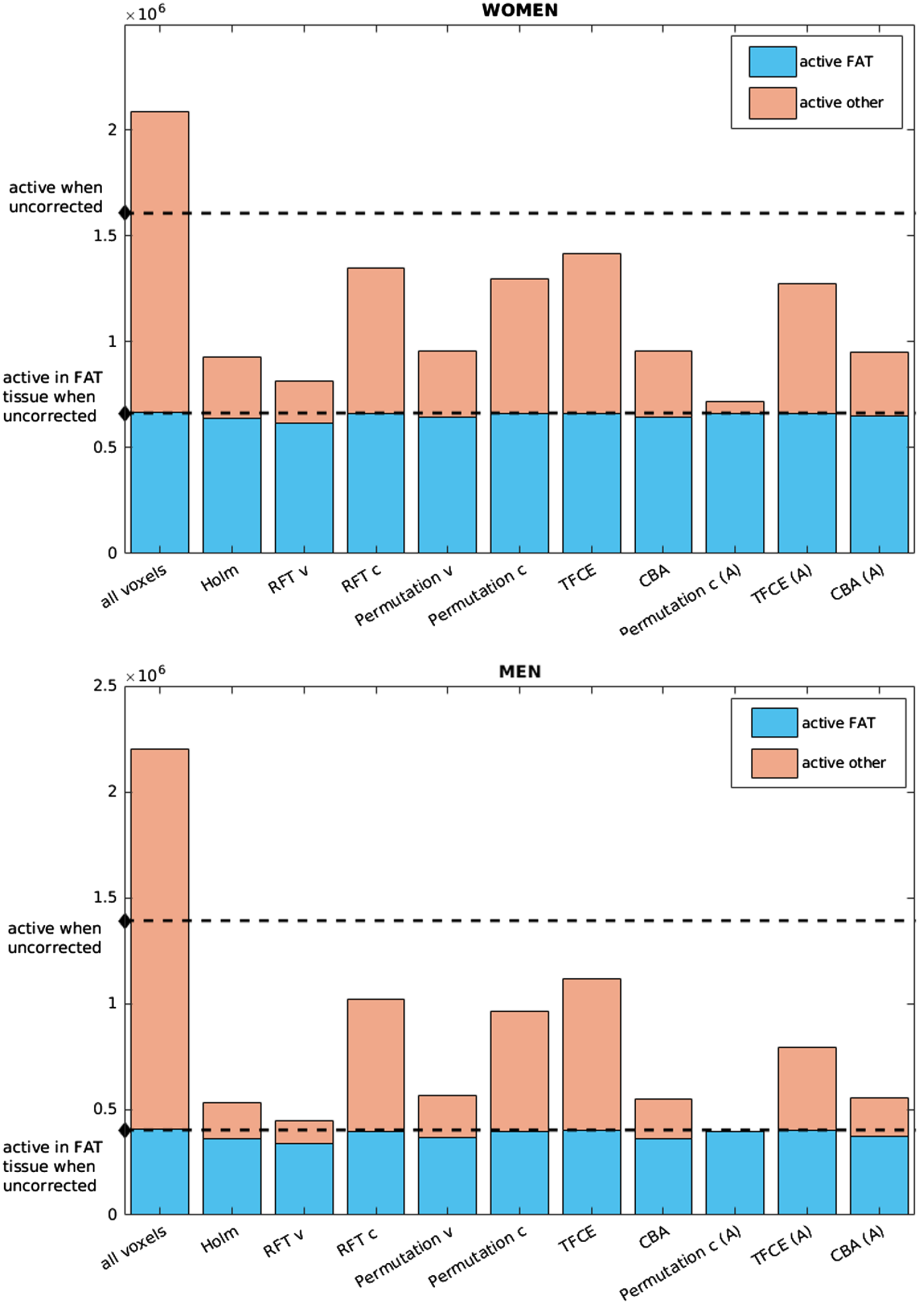
Computing correlation of BIA measurements with JD. The plot shows counts of voxels with activity after each correction, separated by gender. The straight lines represent the uncorrected values. Given the choice of the correlates, we aim for retaining the activity in the fat voxels. All methods marked with (A) are the anatomy-including versions of the original methods.

**Fig. 4:**
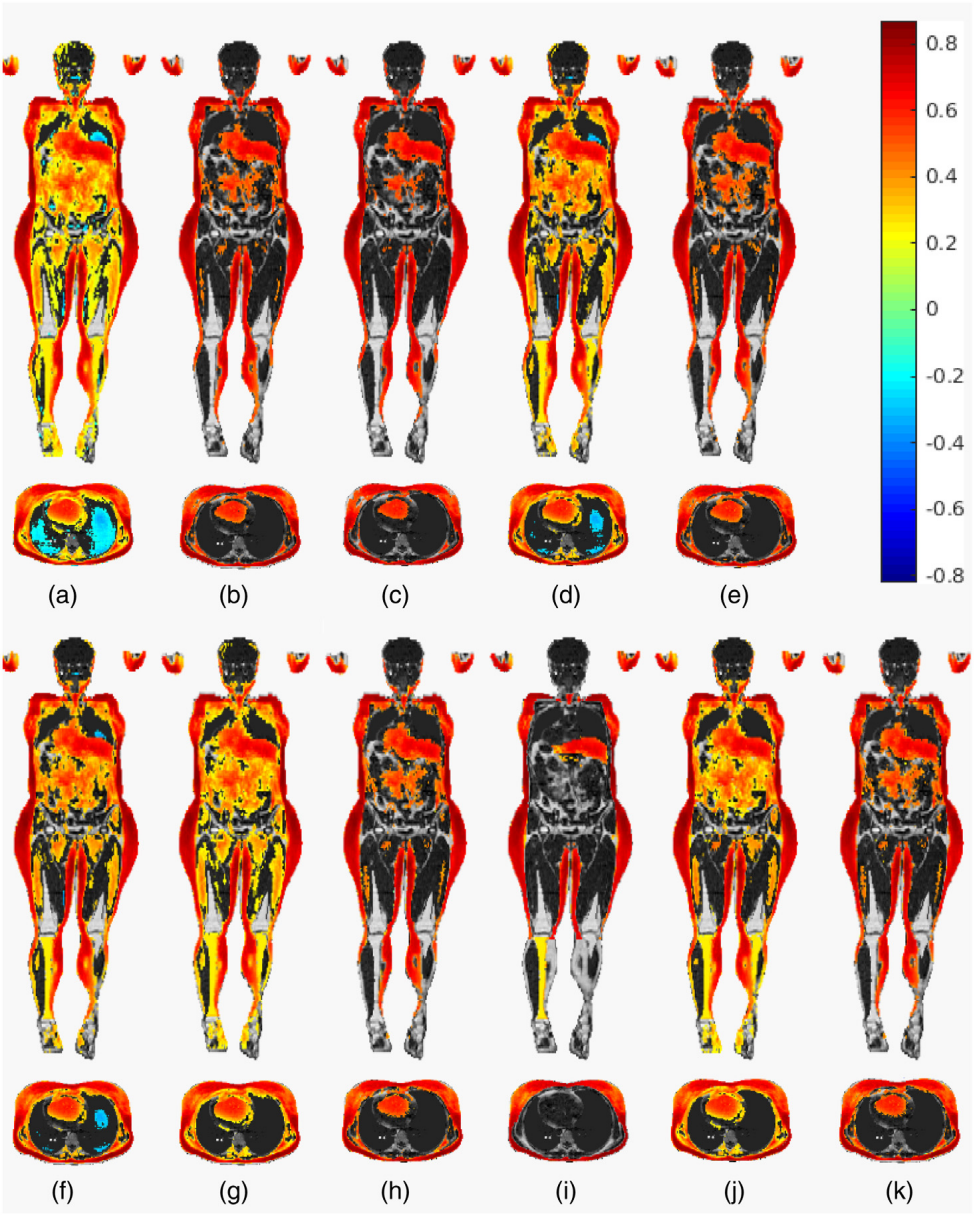
A visual comparison of the effect of correction methods on the BIA measurements versus JD correlation testing for women. Shown here is the reference fat content image, overlayed with the original correlation at voxels that are deemed significant at the significance level α=0.05 under the given multiplicity adjustment. The color corresponds to the strength of the correlation. Each segment (a)–(k) shows a separate method: (a) the original correlation; (b) Holm corrected; (c) RFT voxel- and (d) RFT cluster-wise corrected; (e) and (f) permutation-based voxel and cluster-extent correction, respectively; (g) TFCE corrected; (h) CBA corrected; and finally (i)–(k) the anatomy-inclusive corrections by (i) permutation, (j) TFCE, and (k) CBA.

**Fig. 5:**
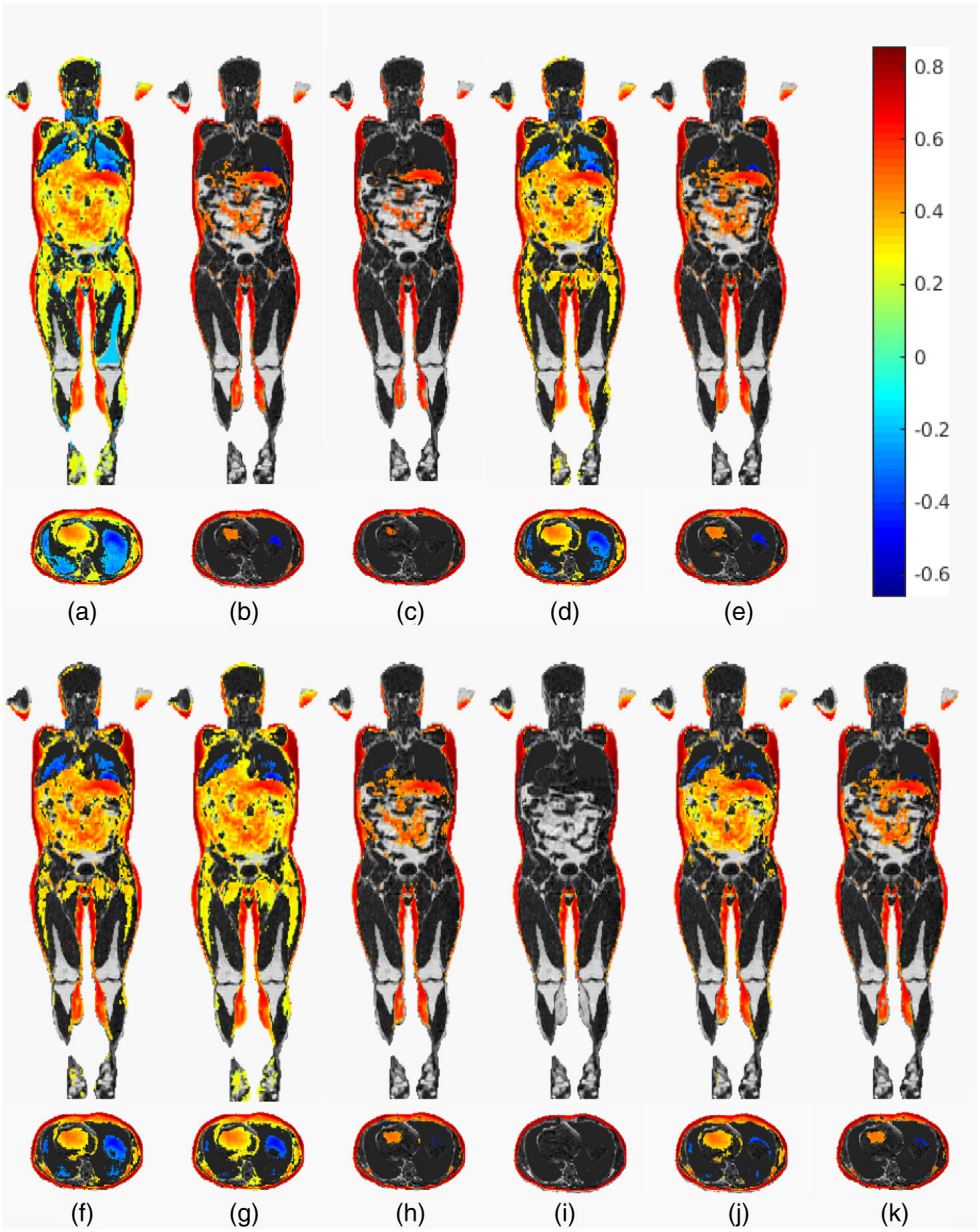
A visual comparison of the effect of correction methods on the BIA measurements versus JD correlation testing for men. Shown here is the reference fat content image, overlayed with the original correlation at voxels that are deemed significant at the significance level α=0.05 under the given multiplicity adjustment. The color corresponds to the strength of the correlation. Each segment (a)–(k) shows a separate method: (a) the original correlation; (b) Holm corrected; (c) RFT voxel- and (d) RFT clusterwise corrected; (e) and (f) permutation-based voxel and cluster-extent correction, respectively; (g) TFCE corrected; (h) CBA corrected; and finally (i)–(k) the anatomy-inclusive corrections by (i) permutation, (j) TFCE, and (k) CBA.

A quantitative comparison of retained activity is shown in Fig. 3, presenting a more detailed view of the voxel counts and method effectiveness. But some of the differences can be noted already from the visual comparisons of example slices in Figs. 4 and 5. Since in the correlation analyses, the effect magnitude and significance become somewhat intertwined, we color the significant voxels by their correlation size to visualize how sensible the significance is.

### 3.2 Evaluation on Null Data

Creating a random correlate vector produces a fair amount of activity due to the large amount of voxels in the body. However, those activities tend to be relatively weak and less clustered. We, therefore, omit example slices, as individual voxel activity is very hard to detect visually from the whole-body image and is not particularly informative either.

**Fig. 6:**
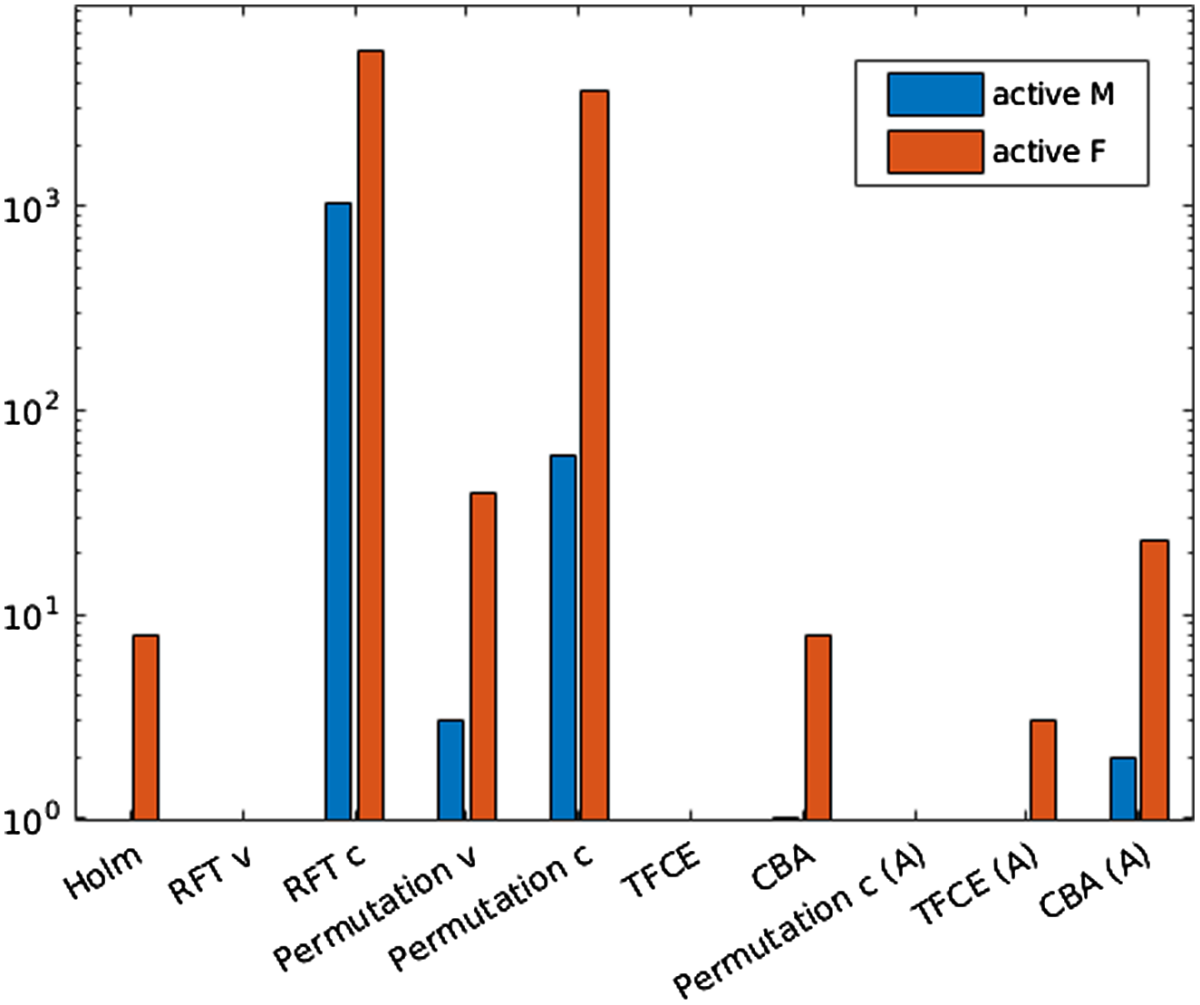
A histogram showing active voxel counts after various corrections, for example, JD versus random vector correlation analysis. Due to extreme imbalance, the counts are drawn in log scale to enable a better comparison.

**Fig. 7:**
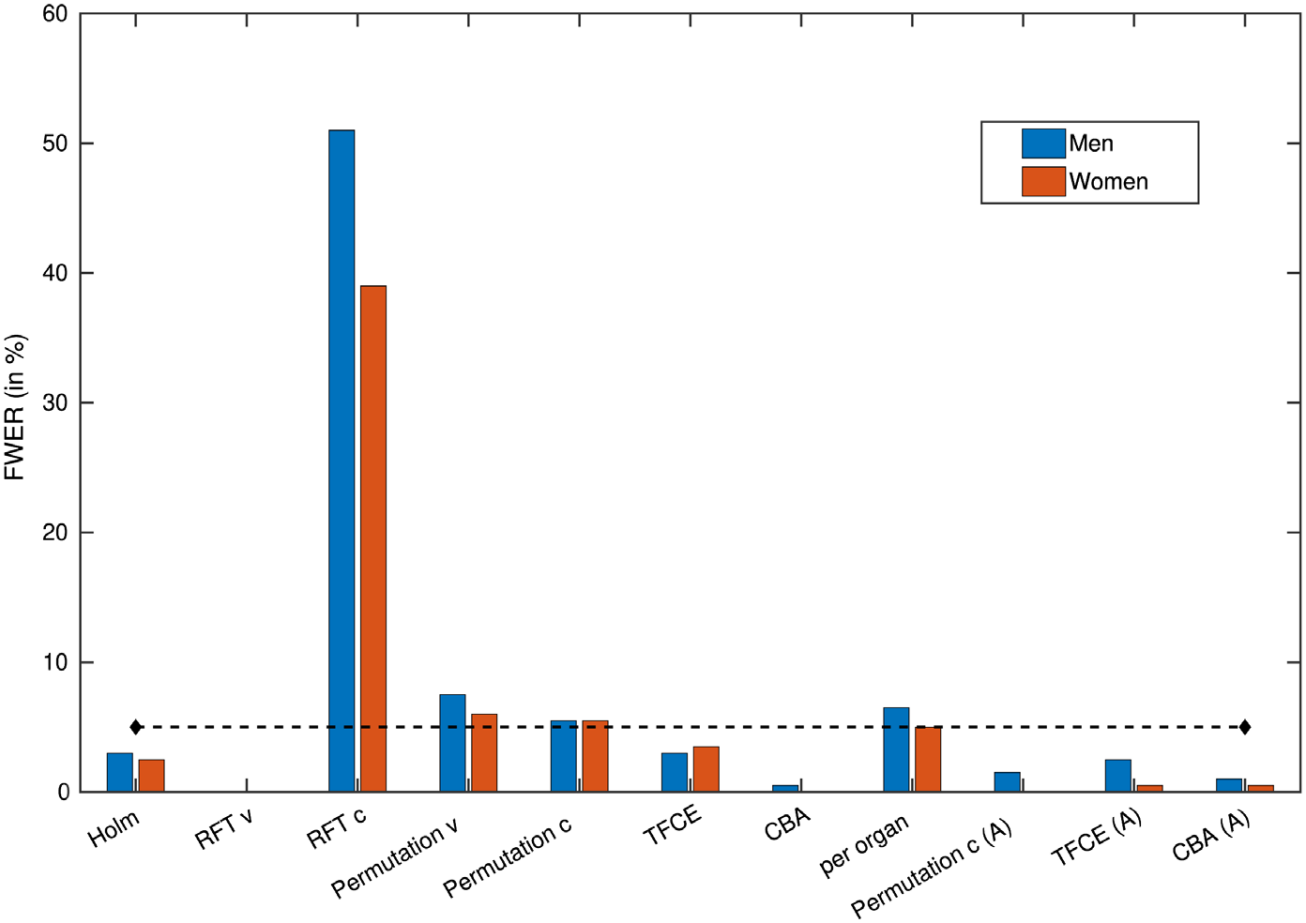
A histogram showing nominal error rates of the methods on 200 random correlate analyses. The values are obtained by dividing the count of runs that had any significance after correction with the number of all runs. The dashed line represents the desired 5% FWER.

To get some insight on how things actually worked, we instead look only at the voxel counts. But since the majority of methods are able to correct for a large extent of those false positive, we show an example log-scale graph of the voxel counts in Fig. 6 to enable proper comparisons. In addition, in Fig. 7, we show the nominal error rates for all the methods, calculated over 200 runs. While that shows the actual rate of error that can be expected over experiments (regardless of the claimed α-error levels), the histogram example can help with understanding the stringency and the extent of failure of the methods.

## 4 Discussion

The histograms in Fig. 3 are intended to highlight the stringency of the methods—ideally, we aim to retain all the observed activity in the high-fat content areas and not much else. However, while the persisting activity in the other areas is some indication on how well the methods dealt with false positives, they cannot be directly used in the argumentation of method choice since there are indications that a certain amount of correlation can be expected also in other tissues (for example, in the lungs^2^).

It is known that the Bonferroni method (as well as Holm and similar) can be disastrously conservative when the test multiplicity is very high. However, as it is visible from Figs. 3–5, a surprisingly large amount of activity persists after correction in the BIA correlation analysis. This is not to say the method is appropriate or encourage its use on the whole body analyses by Imiomics, but rather show that the given analysis produces very high correlations in the fat tissue, which, in turn, means extremely small p values given the high number of subjects. The appropriateness of the other available methods should thus be evaluated with this result (and its theoretical stringency) in mind.

In practice, in neuroimaging, most literature uses a version of RFT-based correction. When resorting to the voxel-based RFT correction, due to the almost inevitable (in imaging) violations of the assumptions, the method behaves highly conservatively, which is confirmed by the results in Figs. 4 and 5: voxelwise RFT appears to be even more stringent than the Holm correction also for whole-body scans and Imiomics analyses.

One of the most obvious violations of the RFT assumptions is the one of smoothness—as the images are already relatively low resolution (with regard to the sizes of various structures we may want to investigate) and the spatial locality is mostly of interest, we cannot afford to apply yet more smoothing as preprocessing. Connected to this is also the highly anisotropic nature of our data. As RFT has been mainly developed for realizations of isotropic random fields, this is another possible source of decreased error control in our applications.

When using the cluster-based corrections, however, the results tend to be much more liberal (see the figures for the results corresponding to RFT and permutation clusterwise correction). This, together with the argument that the activity tends to cluster together even if in very small activity areas, speaks toward doing correction on cluster-extent (or other cluster-involving statistics). But these methods are very sensitive to the choice of the primary threshold and as we can see from the histogram in Figs. 6 and 7, the cluster defining threshold that was used in this evaluation (an equivalent of a p value of 0.001) is still too high to provide sufficient error control under the null hypothesis. Choosing a lower one, on the other hand, would hamper the currently advantageous sensitivity (seen from the BIA analysis).

The TFCE method, while avoiding the arbitrary threshold setting problem, exhibits a surprisingly liberal control in the BIA case (Fig. 3), considering that it generally tends to be more conservative than the methods using an arbitrary cluster defining threshold (unless the chosen threshold is extremely low). At the same time, it keeps a proper control of the nominal error rate, as seen from Fig. 7.

The original and the anatomy compliant versions of the TFCE method appear to have very similar effects on the data and problem at hand, though the anatomy including version is slightly more stringent on the BIA analysis (noticeable even visually in the male subject, Fig. 5). It, however, correctly identifies all the fat voxels, which means its added stringency can be considered an advantage (under the assumption that the fat labeled voxels are the only ones, where activity should be present). The latter holds also for the anatomy-based permutation method.

All of the anatomy-based corrections provide a clearer distinction between active and nonactive areas (Figs. 4 and 5), as they limit them by the underlying tissue extent. The first, per organ-based correction, performs expectedly well on the given example of JD versus BIA correlation, as the activity depends on the tissue type more than its position. However, the extreme loss of spatial locality it suffers renders this method inappropriate for use with any problem, where the activity is more clustered and not regional.

Others among the anatomy including corrections retain the same spatial information as their nonanatomy including counterparts. But they mostly perform better in terms of error control. The reason for that could be that the inclusion of anatomical priors helps to avoid detecting any smeared signal that spans multiple functional areas. In addition, the anatomy-aware permutation method for clusterwise correction effectively limits the maximal size of the clusters (by the size of the maximal tissue extent), meaning that smaller clusters now have a higher chance of turning up as significant, as long as they are a part of a smaller organ.

This additional assumption, that any present activity should be somehow related to the underlying organ extent, is common to both the anatomy-aware versions of the permutation and the TFCE method. The added assumption and an inconvenience of a different meaning of p values do not, however, affect the anatomy-aware CBA and per-organ methods, whose predefined clusters make them more robust and easily interpretable. They do, in turn, require a smart summarizing statistic choice and more problem-specific handling with regard to cluster formation.

Comparing the original correlation map with the results of both versions of the CBA method in Figs. 4 and 5 confirms that the CBA’s clustering produces clusters that are very small and thus pose no hindrance for general visual inspection and inference. However, the clustering is very computationally intense and requires a pilot image that is not used in subsequent analyses, which is perhaps also the reason why the original CBA method has not gained wider use.

A shared disadvantage of all the anatomy-aware methods presented here is the need for a reference semantic segmentation of the underlying tissues and anatomical areas. But they do also share another advantageous property: all anatomy-based versions of cluster-based corrections enable additional parallelization because every anatomical structure can be processed separately. For some of the methods, making them faster would be a large advantage since they take a long time to run in order to obtain reliable results. While all resampling methods already allow for some parallelization due to the independence of different permutations, the CBA method clustering part does not. In the anatomy-based CBA, on the other hand, the clusters can only grow inside the organs so the parallelization using separate anatomical structures is very natural.

Moreover, despite that the original permutation-based (including TFCE) methods can, in theory, run permutations in parallel, this is in practice hard to achieve if dealing with large data since each parallel execution requires a copy of the whole dataset or simultaneous access has to be supported. Both can be problematic or cause delays. But when parallelizing them based on the individual organs, the simultaneously considered datasets are smaller and the image data not shared.

Seeing that the balance between activity retention (Figs. 3–5) and nominal error rates and sizes (Figs. 6 and 7) is more favorable for the anatomically informed approaches, those would be the recommended choice of multiplicity correction methods for whole-body MRI analyses. When higher-level regional activities are of interest, the per-organ correction with appropriate choice of summarizing statistic and segmentation accuracy based weights should be considered, whereas the anatomy-aware TFCE method (preferably with the principled setting of the parameters, as described in Sec. 3) should be used when spatial specificity is needed.

If a segmented reference image is absolutely not available, one can select the original TFCE method or the permutation-based cluster-extent correction. However, being sensitive to the cluster-defining threshold, the latter should be applied with caution and using TFCE instead is advised.

## 5 Conclusion

We presented a number of anatomically oriented multiplicity correction methods and provided an extensive evaluation of various FWER limiting procedures on POEM data and correlation analyses by Imiomics. The inclusion of anatomical structural priors into correction steps is a new idea that has not been widely researched yet. In addition, the provided evaluation complements the available literature on correction methods with imaging data (and analyses) outside the neuroimaging domain.

It may seem that multiple correction problem is not as severe in the whole body analyses as in other applications since the structures (organs) are better defined, and thus, a few incorrectly detected voxels here and there should not be problematic for human observers. But due to a very high number of tests, it is still quite plausible to get falsely detected voxels that are all part of a contiguous region. Therefore, establishing a principled choice of correction method that works well with the whole-body MRI data is very important.

Based on the performed experiments and considerations of method properties, we provided suggestions for the most suitable approaches to be used. While the anatomy-aware methods that have been presented are a step forward to the inclusion of the problem-specific information, there is a lot of space for improvement.

Possible directions for the future work on such improvements include, for example, development of new anatomy-aware methods that avoid the downsides of the currently suggested ones (such as arbitrary choice of method parameters for TFCE) or a detailed investigation into the effects of registration and/or segmentation method choice and accuracy on the correction approaches. Furthermore, when considering including prior (anatomical) information, numerous variations, and hybrids of the herein described methods are possible, depending on what is the preferred interpretation and inference level. Their applicability should also be evaluated.
